# Fast Reduced Graphene-Based Membranes with High Desalination Performance

**DOI:** 10.3390/membranes11110846

**Published:** 2021-10-29

**Authors:** Shanshan Liang, Liuyuan Zhu, Shuai Wang, Liang Chen, Haiping Fang

**Affiliations:** 1 School of Physics, East China University of Science and Technology, Shanghai 200237, China; zhuly666@126.com (L.Z.); wangshuai0008@outlook.com (S.W.); fanghaiping@sinap.ac.cn (H.F.); 2 Department of Optical Engineering, Zhejiang Prov Key Lab Carbon Cycling Forest Ecosy, College of Environmental and Resource Sciences, Zhejiang A&F University, Hangzhou 311300, China; liangchen@zafu.edu.cn

**Keywords:** graphene oxide, membrane, ions transportation, water/ions selectivity

## Abstract

Graphene-oxide (GO) membrane with notable ions sieving properties has attracted significant attention for many applications. However, because of the water swelling of GO membrane, the rejection of monovalent metal cations is generally low. In this work, we developed a fast and facile method to fabricate a kind of reduced GO membranes using the thermal treatment method at 160 °C for only one minute, which denoted as fast reduced GO membrane (FRGO). Surprising, the FRGO membrane represents high ion sieving ability and ultrahigh water/ions selectivity, compared with other reduced GO membranes with similar average interlayer spacings, and even superior to most of GO-based membranes reported in literature. Building on these findings, we provide a new light on fabricating of energy- and environment-related high desalination performance of GO-based membranes as well as a new insight into the transport mechanism within 2D laminar nanochannels.

## 1. Introduction

Membrane technology, as energy-saving and high efficiency technology, has become the predominant choice for desalination and water purification [1,2]. Importantly, graphene, a kind of a typical two-dimensional nanomaterials, has shown great potential in fabricating new-generation membranes benefiting from their unique atomic-thin thickness, robust mechanical strength, and chemical-stable characteristics [3]. Graphene oxide (GO)—a oxygenated derivative of graphene—encompasses abundant oxygen functional groups, such as epoxy, hydroxyl, and carboxyl on the graphene basal plane, making the GO significantly hydrophilic [4]. The stacking of GO nanosheets forms laminated structure and thus creating a lot of nanochannels for efficient mass transport [5]. By benefiting from the above properties, GO-based membranes have emerged as a new platform for desalination [6,7,8,9,10]. 

The transportation of ions and water molecules in GO-based membranes is considered to mainly occur in the massively interconnected nanochannels formed between two neighboring stacked nanosheets; therefore, the controlled sizes of the nanochannels (also known as the interlayer spacings) are of importance for the membrane selectivity and separation properties [11,12,13]. However, in aqueous solution, it is still a great challenge for GO membrane to efficiently restrict swelling, which expands the interlayer spacings and impedes ion sieving by the membranes [14,15,16]. Many attempts had been tried to inhibit the swelling phenomenon via physical (i.e., physical confinements of interlayer spacing by epoxy glue [17] or under internal pressure compaction [18]), and chemical approaches (covalent linking or reduction) [19,20,21] with the aim of precise controlling the interlayer space at sub-nanometer scale to enhance the ion rejection. In 2017, our group provided the cationic-controlled strategy [22] to control interlayer spacings of GO membranes based on cation-π interaction [23,24]. We found that the cations themselves can determine and fix the interlayer spacings of GO membranes at sizes as small as a nanometer and the variable range of this spacing can be controlled to within one ångström.

Researchers have shown that GO membrane after reduction, that is the reduced GO (rGO) membrane, may be a promising way for controlling interlayer spacings [25,26,27]. There are two main methods for fabricating rGO membranes. One is the chemical reduction method [28,29]. For example, HI vapor reduction is a common chemical method to reduce the GO membrane, however, the HI molecules are need to permeate through the back side of the membrane to the front side, thus the reduction degree of the two sides are different [30,31]. UV radiation chemical reduction is also a kind of chemical reduction method, while the thickness of the membranes should be considered because of the penetration of the UV radiation [32,33]. Thermally reduction methods, another main method for rGO membrane, can avoid the above problems [34]. By adjusting the annealing temperature and duration, the degree of the reduction can be tuned, and the interlayer spacings of the rGO membranes are changed, correspondingly [35,36,37]. Generally, the adopted annealing temperature for rGO membranes is above 120 °C over one hour [38]. The fabricated rGO membranes often show good dye rejection while they are not highly efficient for metal ions [39]. Recently, Li et al. attempted a mild condition (80 °C for 25 h) to obtain the more order laminar structure [40]. The disadvantages are a long time lasting of this method and the mild reduction of GO may cause excessive swelling to sieve metal ion. In all, although interlayer spacing constitutes a crucial factor to determine ion transport through the GO-based membranes, the control mechanism and how the controlled interlayer spacings affect the water/ions transportation have not been systematically understood yet, especially for rGO membranes. Thus, more facile and less time-consuming method is still needed to control nanochannel of GO-based membranes. 

In this work, we developed a fast and facile method to reduce GO membrane using 160 °C thermal temperature with only one minute. This rGO membrane is denoted as FRGO membrane. Compared to other rGO with the similar average interlayer spacings, the FRGO membrane represents high ions sieving ability and ultrahigh water/ions selectivity, and even superior to most of GO-based membranes. Further, the underlying mechanism for the GO-based membranes displayed different separation performance is proposed to better understand the transportation of ions and water molecules within the 2D laminar nanochannels.

## 2. Materials and Methods

### 2.1. Preparation of GO Solutions

The GO solutions were prepared using the modified of Hummer methods from natural graphene powder. First, 3 g graphene powder, 2.5 g K_2_S_2_O_8_, and 2.5 g P_2_O_5_ were dissolved in 12 mL H_2_SO_4_, stirred continuously at 80 °C for several hours. Then, the mixture was diluted, centrifuged, and washed with DI water. After drying at 60 °C overnight, preoxidized graphite was obtained. Next, they were further oxidized in concentrated 120 mL H_2_SO_4_, then 15 g of KMnO_4_ powder was added slowly to the solution at constant temperatures, which were 6 °C for GO nanosheets. The solutions were stirred at 35 °C for 2 h and then diluted with 250 mL DI water at 12 °C for GO solutions. The solutions were further stirred at room temperature for 2 h, and then H_2_O_2_ (30%, 20 mL) was added immediately after dilution with 700 mL DI water. The solutions were stirred for 0.5 h, left overnight. Then, centrifuged at 12,000 rpm and washed with a 1:10 HCl aqueous solution and DI water subsequently to remove ions. Few layers of GO were separated using centrifugation again at 4000 rpm. Finally, the GO suspensions were collected and diluted with 1 L DI water, followed by ultrasound. The concentration of as-prepared GO suspensions was ~5 mg/mL.

### 2.2. Fabrications of Different GO Membrane

GO membranes supported by mixed cellulose ester (MCE, 50 mm diameter, 0.22 μm pore size) substrates were fabricated by a vacuum filtration method. 1 mL GO suspensions (5 mg/mL) were diluted into 50 mL DI water, and thus obtained the 0.1 mg/mL GO suspensions. Then, the GO membranes were fabricated by vacuum filtration the 0.1 mg/mL GO suspensions. The rGO membranes were thermally treated using a series of temperatures in an oven for a certain time. The WRGO, MRGO, and FRGO membranes were treated at 80 °C for 24 h, 120 °C for 10 min, and 160 °C for 1 min, respectively. After reduction, the membranes were stored in plastic Petri dishes before membrane tests. These membranes were all used in ions permeation experiment.

### 2.3. Characterization

Membrane surface and cross-sections morphologies were characterized by scanning electron microscope (SEM) (Hitachi, Tokyo, Japan). Before observation, all the samples were sputtered by gold about a thickness of 5 nm, operated at an accelerating voltage of 5 kV. Interlayer spacings were measured by X-ray diffraction (XRD). XRD tests were performed on a Miniflex 600 X-ray diffractometer with Cu Kα radiation (λ = 0.15418 nm) operating at 40 kV, 40 mA (Rigaku, Tokyo, Japan). X-ray photoelectron spectroscopy (XPS) results of the membranes were characterized by the ESCALAB 250XI spectrometer (Thermo Fisher, Waltham, MA, USA). Water contact angles were measured using the sessile drop method on JY-82B (Kruss DSA Hamburg, Germany) at 25 °C. Fourier Transform Infrared spectroscopy (FTIR, Bruker Optics, Karlsruhe, Germany) was used to confirm the functional groups on the three membranes. The zeta potential was analyzed with a NanoBrook 90 Plus PALS instrument (Brookhaven instrument, Holtsville, NY, USA) to characterize the surface charges of the GO/rGO suspensions. All the membranes were at dry state before measured.

### 2.4. Ion Permeation Experiments

Single ion permeation tests were carried out by a self-made device as shown in Figure S1. The effective area of the membrane is 4.71 cm^2^. Then, 80 mL of 0.1 mol/L Na_2_SO_4_ solution was added into the feed side, while 80 mL DI water was added into the permeate side. After free permeation for a certain time, the concentration of Na^+^ in the permeate side was measured by Inductive Coupled Plasma Emission Spectrometer (ICP-OES, Optima 7000DV, Perkin Elmer, Waltham, USA).

The permeation rate of ions (Na^+^) was calculated by using the following equation:

(1) $$P_{i}=\frac{C_{i}\times V}{A\times \Delta t}$$

where C_i_ is the ion concentration in the permeate side after being permeated, V is the solution volume of permeate side, A is effective area of membrane, and Δt is permeate time. The experimental were carried out using the same set-up.

The water permeance was calculated by using the following equation:

(2) $$P_{w}=\frac{\Delta V}{A\times \Delta t}$$

where ΔV is the change volume of feed side, A is effective area of membrane, and Δt is permeate time. The experiments were carried out using the same set-up. 

## 3. Result and Discussion

**Figure 1:**
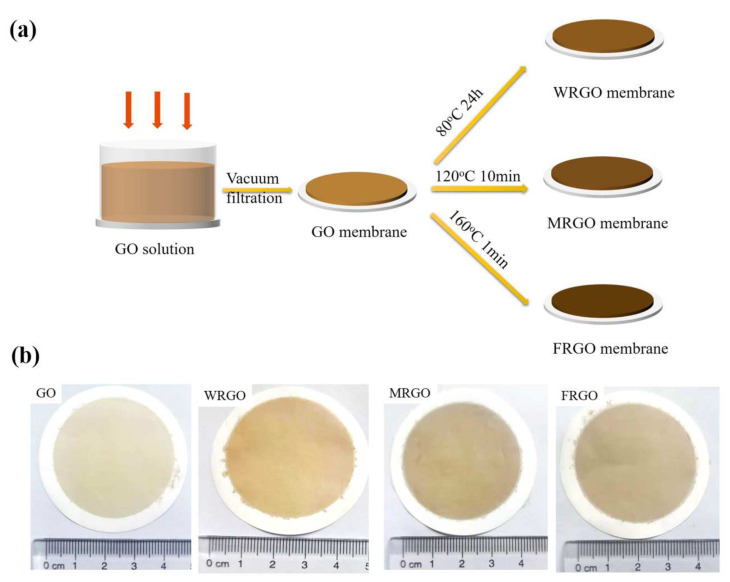
(**a**) Procedure for fabrication and (**b**) photographs of GO membrane, WRGO membrane, MRGO membrane, and FRGO membrane.

As illustrated in Figure 1a, GO membranes were prepared from 80 mL, 5.0 mg/L GO suspensions on mixed cellulose ester (MCE) substrates using vacuum filtration. In order to obtain the same interlayer spacing in the dry state, three different thermal conditions were chosen to reduce the GO membrane. The FRGO membrane was fabricated under the high temperature (160 °C) with only one minute. The MRGO membrane was fabricated under the moderate temperature (120 °C) with 10 min. The WRGO membrane was fabricated under the low temperature (80 °C) with 24 h. The thicknesses of the four membranes are all about 200 nm (see Figure S1 in Supporting Information). 

**Figure 2:**
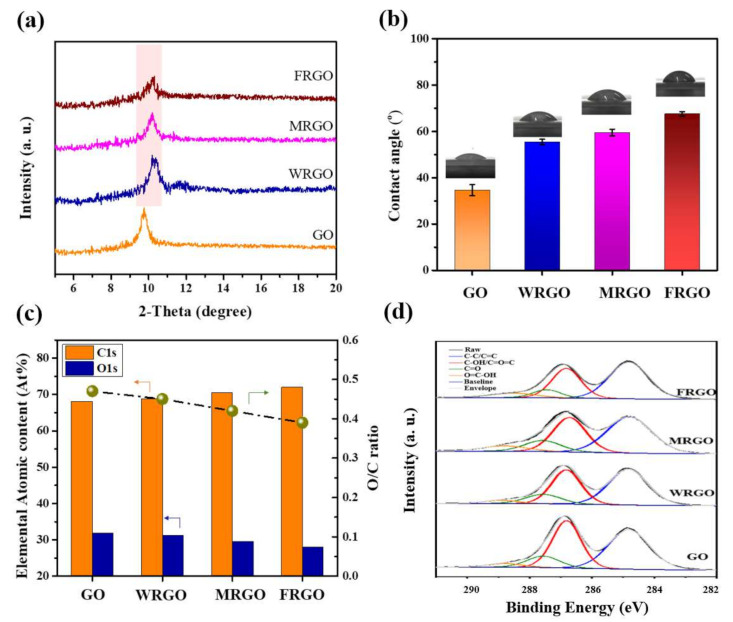
The characteristics of the GO membrane, WRGO membrane, MRGO membrane, and FRGO membrane. (**a**) XRD patterns in the dry state, (**b**) water contact angles, (**c**) elemental atomic contents, and (**d**) XPS spectra of GO membrane, WRGO membrane, MRGO membrane, and FRGO membrane. Detailed spectral fitting and interpretation of the membrane showing raw data (black lines), Envelope lines (grey lines), C-C/C=C peaks (blue lines, 284.8 eV), C-OH/C-O-C peaks (red lines, 286.7 eV), C=O peaks (green lines, 287.6 eV), and O=C-OH peaks (orange lines, 288.8 eV).

As our expected, the interlayer spacings of FRGO membrane, MRGO membrane, and WRGO membrane are very similar in the dry state. In details, the interlayer spacings (indicated the Bragg peaks) of FRGO membrane, MRGO membrane, and WRGO membrane are 8.67 Å, 8.69 Å, and 8.68 Å, respectively (see Figure 2a). In addition, the interlayer spacings of the three rGO membranes are all smaller than the interlayer spacing of GO membrane, which is 9.07 Å in the dry state. These results demonstrate that the interlayer spacings of the GO-based membranes can be critically tuned by different thermal temperature combined with the different holding time. Further, it can be found the XRD Bragg peak of the FRGO membrane is the broadest, followed by that of the MRGO membrane, while that of WRGO membrane is the sharpest, even if the three rGO membranes own similar interlayer spacings. The full width at half maximums (FWHMs) of FRGO membrane, MRGO membrane, WRGO membrane, and GO membrane are 0.463, 0.384. 0.366, and 0.347, respectively. In fact, the interlayer spacings correspond to the largest distribution of the spacings in-between the GO-based membranes [22]. Moreover, the peak intensity of the FRGO membrane is the weakest, indicating that the formation of defects on the graphene basal planes. Thus, unlike MRGO membrane, and WRGO membrane which have narrower nanochannels distributions, the FRGO membrane in the present work has the widest range of the nanochannel size and the decreased order of assembled rGO nanosheets.

Obviously, the color change of the GO-based membranes after thermal reduction can be found. It changes from metallic yellow to brown, as shown in Figure 1b. The darker color is a common phenomenon after reduction the GO-based membranes. It is most likely because the metallic property of graphene is recovered when the graphitic sp^2^-hybridized bonding is restored [41]. However, by comparing the top-view SEM images of GO membrane, FRGO membrane, MRGO membrane, and WRGO membrane (see Figure S2 in Supporting Information), the surface morphologies of the four GO-based membranes are not very different, indicating that the thermal treatment does not destroy the morphologies of the GO-based membranes after reduction [30]. The affinities of the four GO-based membranes are confirmed by the water contact angle tests. The water contact angles of GO membrane, WRGO membrane, MRGO membrane, and FRGO membrane are 34.6°, 55.4°, 59.5°, and 67.7°, respectively. Therefore, the hydrophobic properties of the membrane increase following the order of FRGO membrane > MRGO membrane > WRGO membrane > GO membrane. It can be attributed that more quantities of oxygen functional groups (e.g., carboxylic, epoxide, and hydroxyl) of the nanosheets that compose the membrane are partially eliminated for the FRGO membrane, which will be discussed later [40].

XPS and FTIR spectra were employed to confirm the change of chemical properties of these GO-based membranes. First, XPS characterization was used to determine the ratio of oxygen to carbon of the GO-based membranes, as well as the types and approximate contents of different oxygen-containing functional groups. As shown in Figure 2c and Figure S3 in Supporting Information, in terms of elemental atomic contents of the GO-based membranes obtained from XPS, C1s atomic percent progressively rise with the order of FRGO membrane > MRGO membrane > WRGO membrane > GO membrane, while O1s atomic percent drops in the same order. Overall, the O/C ratios of the GO membrane, WRGO membrane, MRGO membrane, and FRGO membrane are 0.47, 0.45, 0.42, and 0.38, respectively. The C1 s XPS spectrum of the GO-based can be fitted with four components: C-C/C=C (284.8 eV), C-OH/C-O-C (286.7 eV), C=O (287.6 eV), and O=C-OH (288.8 eV) [13,36]. Figure 2d shows the detailed spectral fitting and interpretation of the GO-based membranes. It can be found that the oxygen-containing functional groups in the FRGO membrane are in the smallest proportions compared with other GO-based membranes, especially for the C-O bond. In other words, for FRGO membrane, the degree of elimination of the oxygen functional groups is the biggest. The results demonstrate that the distributions of the oxygen functional groups from the GO laminates are tunable based on the different thermal treatment, although the interlayer spacings are very similar. The similar evidence can be provided by FTIR.

According to FTIR spectra (see Figure S4 in Supporting Information) of FRGO membrane, the-OH (hydroxyl) stretching vibration at ~3390 cm^−1^ is notable diminished while the C-O stretching (mainly COOH) almost remains unchanged, indicating that for FRGO membrane, the COOH could not effectively be removed under this fast thermal reduction condition. Further, compared to FTIR spectra of GO membrane, the epoxy (C-O-C) groups peak at 1220 cm^−1^ increases. It can be attributed to the esterification reaction within the neighboring GO nanosheets. The other two reduced GO membranes (WRGO membrane, and MRGO membrane) also present the similar trend to some extent. In all, the above results can be deduced that the reduction degree of FRGO membrane is the highest, even though the thermal treatment time for FRGO membrane is very fast (only one minute).

Zeta potential is a vital indicator of stability for dispersion system. It can be seen from Figure S5 in Supporting Information that zeta potentials of GO, WRGO, MRGO, and FRGO suspensions are −51.32, −56.07, −61.52 and −53.37 mV, respectively. The results indicate that the stability of the DI with order of GO membrane < WRGO membrane < MRGO membrane < FRGO membrane.

**Figure 3:**
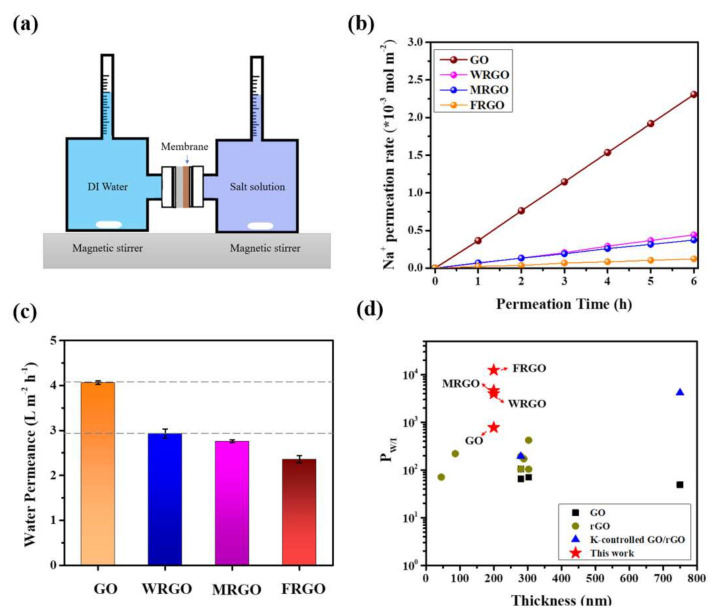
Ion permeation experiments. (**a**) The schematic of the ion permeation experiments, (**b**) the Na^+^ permeation rates, and (**c**) water permeances through GO membrane, WRGO membrane, MRGO membrane, and FRGO membrane. Error bars indicate the standard deviation from three different samples. (**d**) P_w/I_ comparison with other GO-based membranes (see details in Table S1 in Supporting Information).

Ion permeation tests through the four GO-based membranes with different reduction degree had been investigated using a home-made H-type diffusion cell with two chambers, one containing 100 mL deionized water, and the other containing 100 mL 0.1 mol/L sodium sulfate (Na_2_SO_4_) solution, respectively. The permeation experiment schematic and the osmosis device are shown in Figure 3a and Figure S6 in Supporting Information, respectively. During the test, the GO-based membranes were facing to the draw side. Magnetic stirrings were applied to both sides of the feed and the draw solutions to avoid possible concentration gradients [42]. ICP-OES was used obtain the cation concentrations in solutions.

Figure 3b presents the Na^+^ permeation rates through FRGO membrane, MRGO membrane, WRGO membrane, and GO membrane within 6 h. The permeation rates were calculated based on the amounts of ions penetrating through the GO-based membranes and were extrapolated from the concentration changes in deionized water over time. The plots show that the permeation rates through the membranes follow the order of FRGO membrane > MRGO membrane > WRGO membrane > GO membrane. Since GO membranes swelling in water, the GO membrane itself has weak barrier to Na^+^, so that Na^+^ permeation rate through GO membrane is as high as 0.2168 mmol m^-2^ h^-1^. In contrast, the Na^+^ permeation rates through the three rGO membrane significantly decrease more than an order of magnitude, due to narrower interlayer spacings after thermal treatment as above mentioned. Among them, the Na^+^ permeation rates through MRGO membrane and WRGO membrane are close, that is 0.041 mmol m^-2^ h^-1^, and 0.033 mmol m^-2^ h^-1^, respectively. In special, the Na^+^ permeation rate through FRGO membrane is the lowest, which is 0.011 mmol m^-2^ h^-1^, only about 1/20 of that through GO membrane. It demonstrates the ion sieving effect of the FRGO membrane is better than the other two rGO membranes. 

Next, the stability tests of the four membranes were conducted. It can be found that, for 48 h, the three rGO membranes, the permeation rates are almost the same, and maintain the order of WRGO membrane > MRGO membrane > FRGO membrane (see Figure S7 in Supporting Information), indicating the good stability of these rGO membranes, especially for FRGO membrane. Additionally, the low permeation rates also indicate the integrity of the three rGO membranes, thus they are all have good mechanical stability [28], while for GO membrane, the permeation rate is stable for the first 16 h, but it has an obvious increase after 24 h. It can be attributed the swelling of GO membrane [15]. For a long time, the interlayer spacings are enlarged, thus more ions can permeate through the membrane, and thus the permeation rate is increased.

To elucidate the effect of the initial concentration in the draw side on the ions permeation rate, we chose five concentrations of the Na^+^ as draw solution, that is 0.05, 0.1, 0.25, 0.5, and 1 mol/L. The results are shown in Figure S8 in Supporting Information. It can be clearly found that, the Na^+^ permeation rates increase with the increasing the concentration. It can be attributed the high driving force of the higher concentration, thus leading to a fast permeation rate. On the other hand, for each concentration, the Na^+^ permeation rates through the membranes all follow the order of GO membrane > WRGO membrane > MRGO membrane > FRGO membrane, indicating that FRGO membrane exhibits high ions rejection even at high concentration. 

In addition, the permeation rates of 0.1 mol/L Mg^2+^ through these GO-based membranes were also tested. Similarly, the Mg^2+^ permeation rates through the membranes also follow the order of FRGO membrane > MRGO membrane >WRGO membrane > GO membrane, as the order of Na^+^ permeation rates through the membranes (see Figure S9 in Supporting Information). However, the permeation rates of Mg^2 +^ are almost one order of magnitude smaller than the permeation rates of Na^+^ when through the same membrane. It is because the hydrated diameter of Mg^2+^ is much larger than the hydrated diameter of Na^+^ [12].

Water permeance is another important indicator of GO-based membranes. The water permeances through GO membrane, WRGO membrane, MRGO membrane and FRGO membrane are 4.0 L m^-2^ h^-1^, 2.98 L m^-2^ h^-1^, 2.76 L m^-2^ h^-1^, and 2.46 L m^-2^ h^-1^, respectively, as shown in Figure 3c. It can be found that the water permeances of the WRGO membrane, MRGO membrane, and FRGO membrane slightly decrease with the increasing reduction degree. Even though, water permeance for FRGO membrane still maintains at high level, due to the remaining oxygen groups of the FRGO nanosheets [43]. 

In order to better investigate the selectivity of water molecules and metal ions, the permeation ratio of water and metal ions (P_W/I_) was calculated as follows:

(3) $$P_{W/I}=\frac{water\;permeation\;rate\;\left(mol\cdot m^{-2}\right)}{metal-ion\;permeation\;rate\;\left(mol\cdot m^{-2}\right)}$$

Typically, the P_w/I_ of the FRGO membrane is as high as 12,425. This ultrahigh water/ions selectivity can be attributed to the combined effect of the lowest ion permeation rate and the relative higher water permeance of the FRGO membrane. Thus, compared with other GO-based membranes, FRGO membrane presents better water/ions selectivity at the same thickness, as shown in Figure 3d (see details in Table S1. Supporting Information).

**Figure 4:**
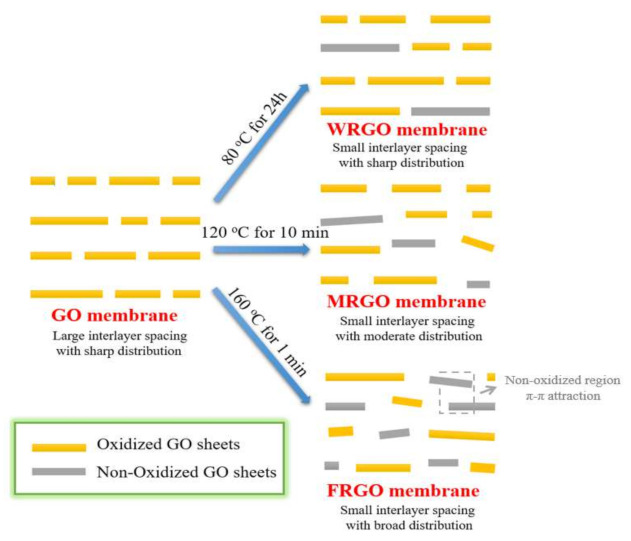
The schematic illustration of the nano-structures of the GO membrane, WRGO membrane, MRGO membrane, and FRGO membranes under the different thermal reduced treatment conditions.

On the basis of the above results, the structural model of the three rGO membrane is proposed in Figure 4. In this mode, it can be found although the average interlayer spacings are similar, the distributions of the nanochannels sizes are different. We believe that the ions transportation in-between FRGO membrane is impeded by the most disordered nanosheets, leading to the lowest permeation rate. In addition, the FRGO membrane has more non-oxidized zones, and water molecules cannot easily pass through the non-oxidized zones due to the π-π attraction force. However, because of the remaining oxygen groups of the FRGO nanosheets, water permeance of FRGO membrane still maintains at high level. The above factors lead to the high water/ion selectivity of FRGO membrane. Overall, the permeation rates of ions and transportation of water molecules can be clearly tuned by adjusting reduction condition by changed the nanochannels structures and distributions, even the average interlayer spacings are very similar.

## 4. Conclusions

In summary, we developed a fast and facile method to reduced GO membrane using 160^o^C thermal temperature with only one minute, that is FRGO membrane. Compared to the GO-based membranes with the similar average interlayer spacing, the FRGO membrane represents high ions sieving ability and ultrahigh water/ions selectivity, and even superior to most of GO-based membranes in literature. Further, the possible mechanism for the GO-based membranes displayed different separation performance is proposed. The ultrahigh water/ions selectivity can be attributed to the co-effect of the lowest ion permeation rate and the relative higher water permeance. These findings help in developing future strategies for designing high water permeance and high water/ions selectivity rGO membranes, which are ideal for an FO process. Overall, our findings reveal the beauty of GO-based membranes in the desalination field ions rejection, and represent a facile step toward ultrahigh water permeance, effective ions rejection, and stable performance GO-based membranes for water treatment.

## Supplementary Materials

The following are available online at http://www.mdpi.com/xxx/s1/. Figure S1: Cross-sectional SEM images of (a) GO membrane, (b) WRGO membrane, (c) MRGO membrane, and (d) FRGO membrane. Figure S2: Surface SEM images of (a) GO membrane, (b) WRGO membrane, (c) MRGO membrane, and (d) FRGO membrane. Figure S3: XPS spectra of (a) GO membrane, (b) WRGO membrane, (c) MRGO membrane, and (d) FRGO membrane over a wide scanning range. Figure S4: FTIR spectra of GO membrane, WRGO membrane, MRGO membrane, and FRGO membrane. Figure S5: Zeta potential analysis of GO membrane, WRGO membrane, MRGO membrane, and FRGO membrane. Figure S6: Photograph of a two-chamber diffusion cell. The H-type cell consists of feed and permeate sides; the membrane is located between them. Table S1: Comparison with other GO-based membranes. Figure S7: The long term of ion permeation rates through GO membrane, WRGO membrane, MRGO membrane, and FRGO membrane. Figure S8: The effect of ions concentrations in the draw solution on membrane permeability. Figure S9: The Mg^2+^ ion permeation rates through GO membrane, WRGO membrane, MRGO membrane, and FRGO membrane.
