# Supplementary material for: Fast Reduced Graphene-Based Membranes with High Desalination Performance
Source: Membranes (Basel). 2021 Oct 29;11(11):846. doi: 10.3390/membranes11110846 (PMC8619719; doi:10.3390/membranes11110846)
Supplement: Supplementary file 1 [file membranes-11-00846-s001.zip › membranes-1301881-supplementary.pdf]

Supporting Information

Fast Reduced Graphene-Based Membranes with High Desalination Performance

Shanshan Liang [1,*], Liuyuan Zhu [1], Shuai Wang [1], Liang Chen [2] and Haiping Fang [1]

[1] School of Physics, East China University of Science and Technology, Shanghai 200237, China; zhuly666@126.com (L.Z.); wangshuai0008@outlook.com (S.W.); fanghaiping@sinap.ac.cn (H.F.)

[2] Department of Optical Engineering, Zhejiang Prov Key Lab Carbon Cycling Forest Ecosy, College of Environmental and Resource Sciences, Zhejiang A&F University, Hangzhou 311300, China; liangchen@zafu.edu.cn

* Correspondence: liangshanshan@ecust.edu.cn

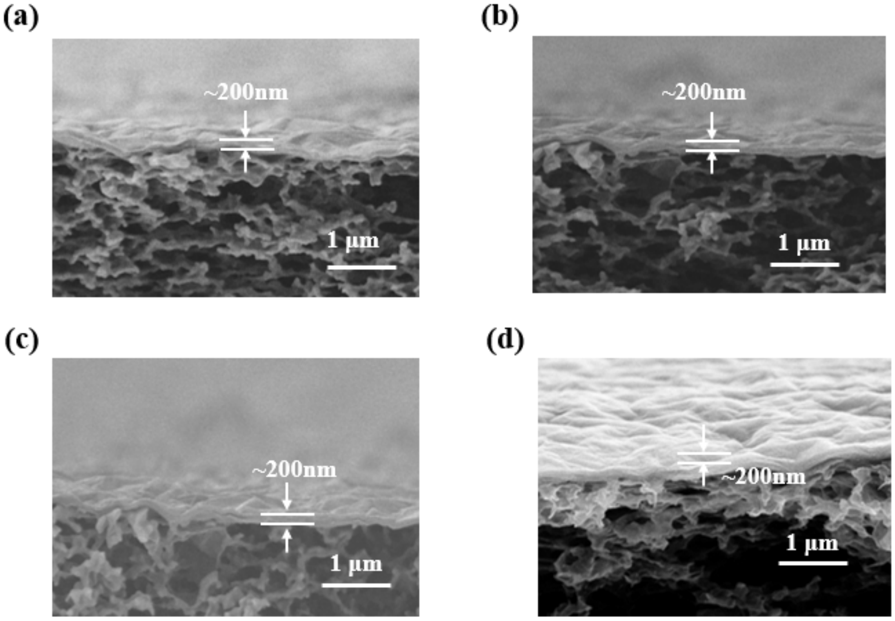

Figure S1. Cross-sectional SEM images of (a) GO membrane, (b) WRGO membrane, (c) MRGO membrane and (d) FRGO membrane.

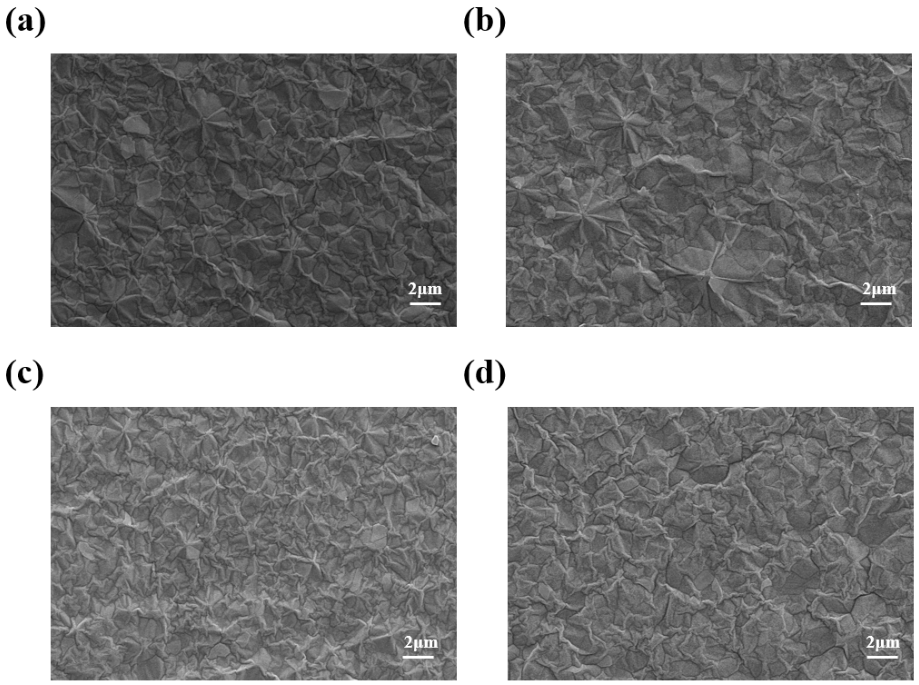

Figure S2. Surface SEM images of (a) GO membrane, (b) WRGO membrane, (c) MRGO membrane, and (d) FRGO membrane.

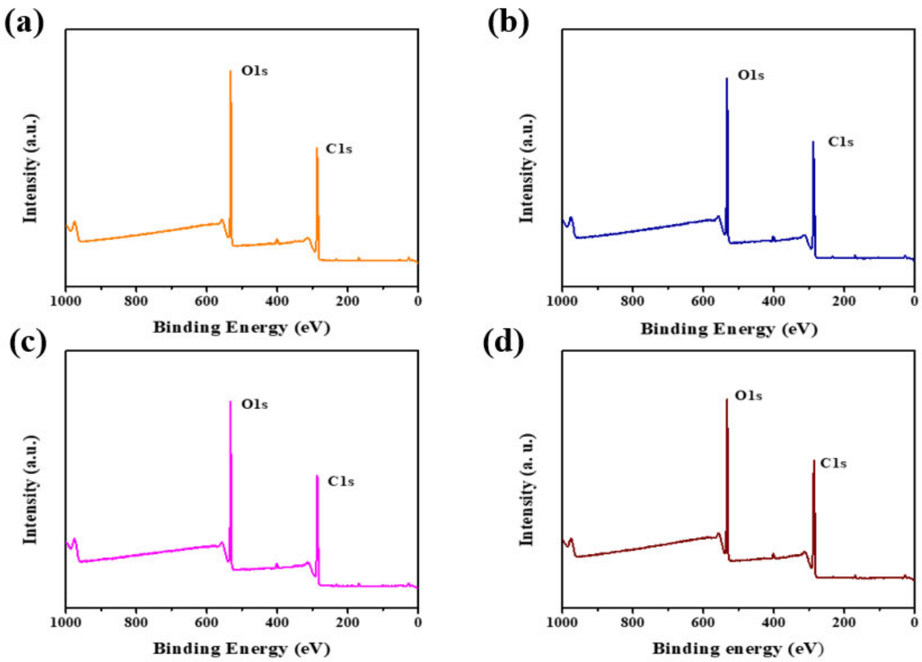

Figure S3. XPS spectra of (a) GO membrane, (b) WRGO membrane, (c) MRGO, and (d) FRGO membrane over a wide scanning range.

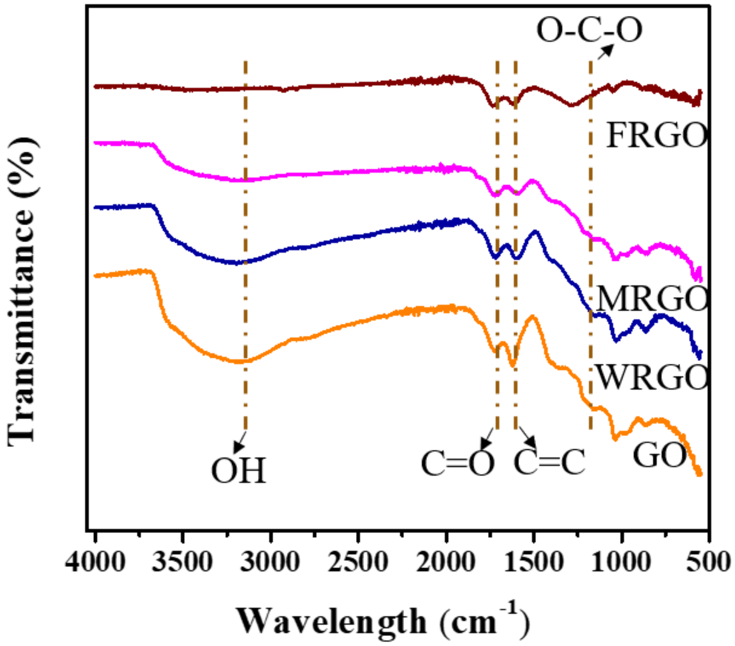

Figure S4. FTIR spectra of GO membrane, WRGO membrane, MRGO membrane, and FRGO membrane.

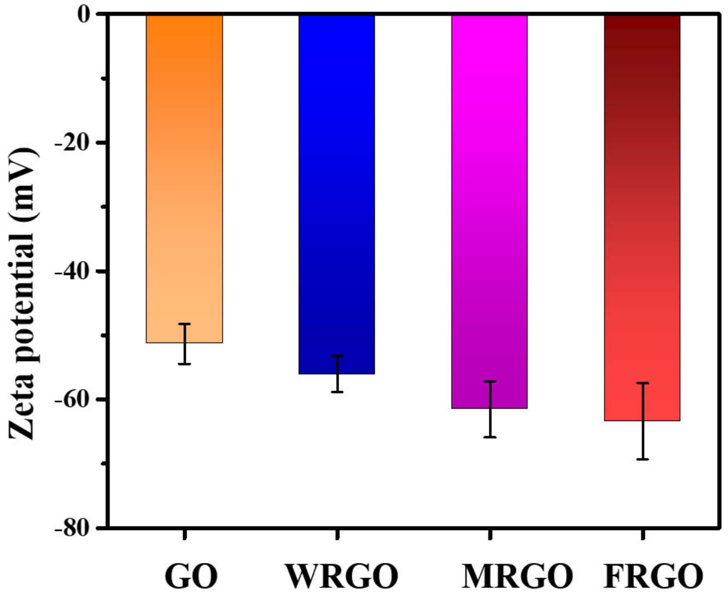

Figure S5 Zeta potential analysis of the GO membrane, WRGO membrane, MRGO membrane, and FRGO membranes.

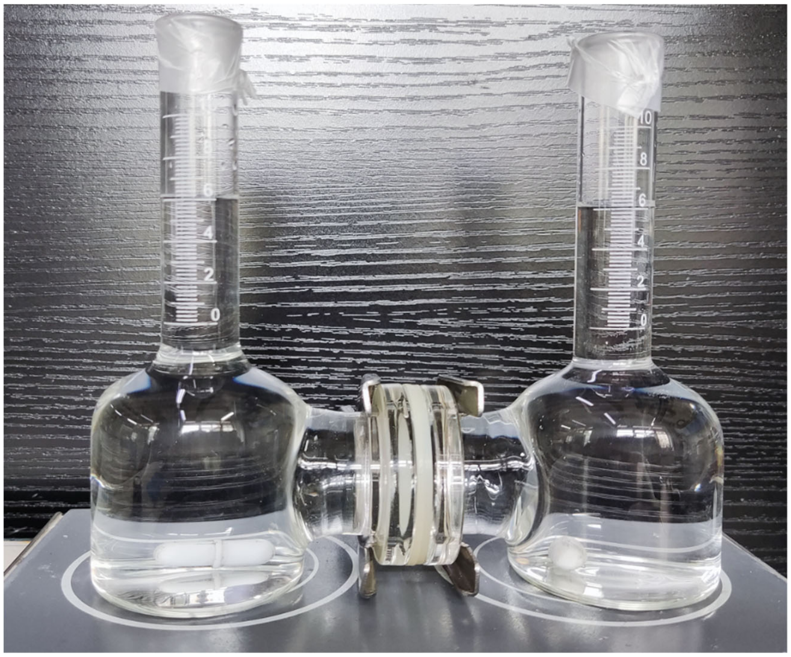

Figure S6. Photograph of a two-chamber diffusion cell. The H-type cell consists of feed and permeate sides; the membrane is located between them.

Supplementary Table S1. Comparison with other GO-based membranes.

Membrane type	Thickness	Na^+ permeation rate (mmol m^{-2} h^{-1})	Water Permeance (L m^{-2} h^{-1})	$P_{w/I}$
GO[S3]	280	0.7184	0.85	65
GO [S4]	303	0.52	0.66	70
GO [S3]	750	0.19	0.17	49
rGO [S3]	280	0.301	0.57	105
K-controlled GO [S3]	750	0.0048	0.36	4166
K-controlled RGO [S3]	280	0.0629	0.22	194
NH_3-rGO [S4]	303	0.054	0.41	422
HI-rGO [S4]	303	0.0059	0.011	104
0.4-R [S5]	289	9.8	30.14	171
1.2-R [S5]	86.5	6.9	86.5	220
2.0-R [S5]	45.4	1.13	1.45	71
GO	~200	0.217	4.04	1035
WRGO	~200	0.041	2.76	3970
MRGO	~200	0.033	2.93	4646
FRGO	~200	0.011	2.46	12425

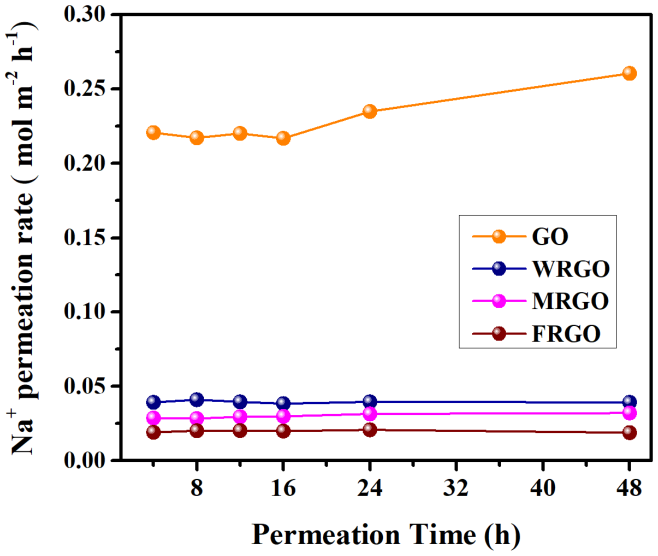

Figure S7. The long term of ion permeation rates through GO membrane, WRGO membrane, MRGO membrane, and FRGO membrane.

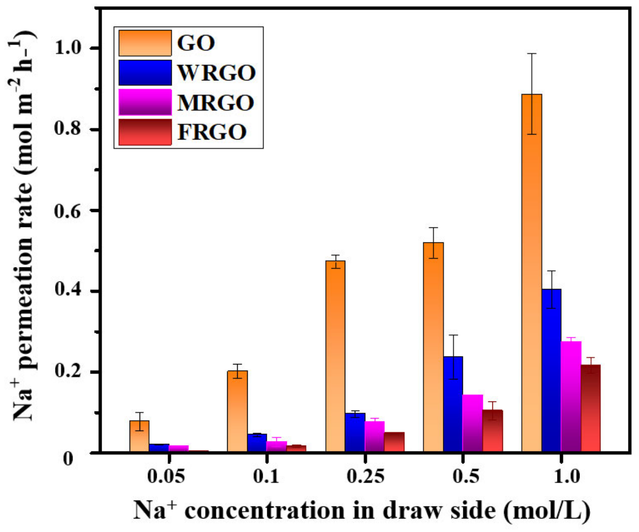

Figure S8. The effect of ions concentrations in the draw solution on membrane permeability.

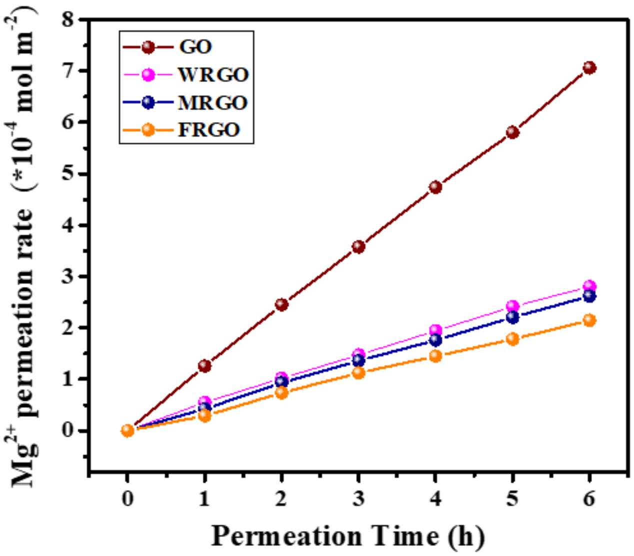

Figure S9. The Mg^{2+} ion permeation rate through GO membrane, WRGO membrane, MRGO membrane, and FRGO membrane.

References

1. Liang, S. et al. *Sep. Purif. Techn*. **2020**, *241*,116738.

2. Dai. F., et al. *Chem. Commun.* **2020**, *56* (95), 15069-15071.

3. Chen, L., et al. *Nature*. **2017**,*550*, 380-383.

4. Zhao, Z, et al, *ACS Sustainable Chem. Eng.* **2019**, *7*(17), 14874-14882.

5. Wang Q., et al, *ACS Appl. Mater. Inter*. **2017**, *9*, 22509-22517.
